# Generation of a new *Adar1p150*^−*/*−^ mouse demonstrates isoform-specific roles in embryonic development and adult homeostasis

**DOI:** 10.1261/rna.079509.122

**Published:** 2023-09

**Authors:** Zhen Liang, Ankita Goradia, Carl R. Walkley, Jacki E. Heraud-Farlow

**Affiliations:** 1 St. Vincent's Institute of Medical Research, Fitzroy, Victoria 3065, Australia; 2 Department of Medicine, Eastern Hill Precinct, Melbourne Medical School, University of Melbourne, Fitzroy, Victoria 3065, Australia

**Keywords:** RNA modification, A-to-I RNA editing, ADAR1 isoforms, innate immunity, type I interferon, ADAR1p150

## Abstract

The RNA editing enzyme adenosine deaminase acting on RNA 1 (ADAR1) is an essential regulator of the innate immune response to both cellular and viral double-stranded RNA (dsRNA). Adenosine-to-inosine (A-to-I) editing by ADAR1 modifies the sequence and structure of endogenous dsRNA and masks it from the cytoplasmic dsRNA sensor melanoma differentiation-associated protein 5 (MDA5), preventing innate immune activation. Loss-of-function mutations in *ADAR* are associated with rare autoinflammatory disorders including Aicardi–Goutières syndrome (AGS), defined by a constitutive systemic up-regulation of type I interferon (IFN). The murine *Adar* gene encodes two protein isoforms with distinct functions: ADAR1p110 is constitutively expressed and localizes to the nucleus, whereas ADAR1p150 is primarily cytoplasmic and is inducible by IFN. Recent studies have demonstrated the critical requirement for ADAR1p150 to suppress innate immune activation by self dsRNAs. However, detailed in vivo characterization of the role of ADAR1p150 during development and in adult mice is lacking. We identified a new ADAR1p150-specific knockout mouse mutant based on a single nucleotide deletion that resulted in the loss of the ADAR1p150 protein without affecting ADAR1p110 expression. The *Adar1p150*^−*/*−^ died embryonically at E11.5–E12.5 accompanied by cell death in the fetal liver and an activated IFN response. Somatic loss of ADAR1p150 in adults was lethal and caused rapid hematopoietic failure, demonstrating an ongoing requirement for ADAR1p150 in vivo. The generation and characterization of this mouse model demonstrates the essential role of ADAR1p150 in vivo and provides a new tool for dissecting the functional differences between ADAR1 isoforms and their physiological contributions.

## INTRODUCTION

Adenosine-to-inosine (A-to-I) editing is one of the most prevalent RNA modifications in mammals. Tens of thousands to millions of A-to-I editing sites have been identified in mammalian genomes, the vast majority of which lie within repetitive elements such as SINEs and LINEs, as well as a smaller number present in coding regions and other RNAs such as miRNAs (Bazak et al. 2014; Picardi et al. 2015; Tan et al. 2017; Pfaller et al. 2018; Licht et al. 2019b). Two catalytically active ADAR enzymes have been identified, adenosine deaminase acting on RNA 1 (ADAR1) (gene: *ADAR*) and adenosine deaminase acting on RNA 2 (ADAR2) (gene: *ADARB1*). When A-to-I editing occurs in a coding region, the physiological functions of the resultant protein may be altered because inosine is usually interpreted as guanosine by the ribosome (Basilio et al. 1962; Martin et al. 1985; Licht et al. 2019a). Recoding of the transcript of the glutamate receptor subunit *GRIA2*, at a single adenosine, is the essential physiological function of ADAR2 in mice (Higuchi et al. 1993, 2000). In contrast, the primary function of ADAR1 editing is to modify the structure and immunogenicity of cellular double-stranded RNA (dsRNA) (Walkley and Li 2017; Eisenberg and Levanon 2018). Alterations in ADAR1 expression or function have been linked to various pathological states, from common autoimmune diseases to cancers (Jain et al. 2019; Li et al. 2022). A subset of Aicardi–Goutières syndrome (AGS), a severe pediatric autoinflammatory disease and type I interferonopathy, are caused by inherited *ADAR* mutations (Rice et al. 2012; Crow and Stetson 2022). In AGS, *ADAR* mutations are most often a compound heterozygote, with a frequent pattern being where one allele has a mutation impacting the p150 isoform (e.g., p.P193A) and a second mutation impacting the deaminase domain or leading to loss of the ADAR1 protein (Rice et al. 2012, 2017). Delineating the full spectrum of ADAR1 functions is critical to understanding the role of each isoform in disease and developing new therapeutics.

The cellular cytosolic antiviral system can distinguish endogenous (“self”) from foreign (“nonself”) nucleic acid (Schlee and Hartmann 2016). An established function of ADAR1 is to mark endogenous dsRNA as “self” through A-to-I editing, thus masking dsRNA from the cytosolic RNA-sensing receptor melanoma differentiation-associated protein 5 (MDA5) (Mannion et al. 2014; Liddicoat et al. 2015; Pestal et al. 2015). Activation of MDA5 and its downstream effector, mitochondrial antiviral signaling protein (MAVS), triggers type I interferon (IFN) production and signaling which initiates a cascade of transcription of interferon-stimulated genes (ISGs). *Adar1* null (*Adar1*^−*/*−^) animals are embryonic lethal between E11.5 and E12.5, accompanied by activated type I IFN, ISG production and cell death across different organs, most notably failed hematopoiesis in the fetal liver (FL) (Hartner et al. 2004, 2009; Wang et al. 2004). Mice expressing an editing deficient ADAR1 protein (*Adar1*^*E861A/E861A*^) also die embryonically at E13.5 with an elevated ISG signature and cell death in the FL (Liddicoat et al. 2015). Deleting either MDA5 or MAVS can rescue the embryonic death of *Adar1* null animals to 2–3 d post-birth (Mannion et al. 2014; Liddicoat et al. 2015; Pestal et al. 2015; Bajad et al. 2020), and more strikingly, can allow survival of ADAR1 editing deficient mice to adulthood (Liddicoat et al. 2015). *Adar1^E861A/E861A^Ifih1*^−*/*−^ mice survive long-term (Heraud-Farlow et al. 2017; Chalk et al. 2019). These genetic studies indicate the importance of ADAR1 protein and A-to-I editing in suppressing the MDA5 sensing pathway and preventing embryonic abnormalities. Importantly, the genetic pathways are conserved in humans. Loss of ADAR1 in human cells triggered an MDA5-dependent up-regulation of the type I IFN response (Pestal et al. 2015; Chung et al. 2018; Pfaller et al. 2018). Consistent with the genetics resolved in mouse models, patients with loss-of-function mutations in *ADAR* or gain-of-function mutations in *IFIH1* (encoding MDA5) both develop AGS (Crow and Stetson 2022).

In both humans and mice, the *ADAR* gene encodes two protein isoforms with distinct functions. ADAR1p110 is constitutively expressed and restricted to the nucleus, where it edits RNA cotranscriptionally (Hsiao et al. 2018). ADAR1p150 is primarily expressed in the cytoplasm and is inducible in response to stimuli such as viral infection and type I IFN (Samuel 2011, 2019). In some organs of the developing mouse, such as the spleen and thymus, it appears to be the predominant isoform (Kim et al. 2021). ADAR1p150 has a unique Z-DNA/RNA-binding alpha domain (Zα) at its amino terminus that can bind to and interact with the alternative left-handed conformation of RNA (Z-RNA) (Herbert et al. 1995, 1998; Nakahama and Kawahara 2021). The importance of understanding the specific functions of ADAR1p150 has been highlighted with rising interest in functions of the Zα domain, particularly as this domain is a mutational hotspot in patients with AGS6 (Rice et al. 2017). Recent studies have interrogated the importance of this domain in repressing the type I IFN response (de Reuver et al. 2021; Guo et al. 2021; Nakahama et al. 2021). In mammals, the Zα domain is found in only one other protein, Z-DNA-binding protein 1 (ZBP1). Some ADAR1 Zα mutant mice have RIPK3-dependent necroptosis and inflammation triggered by ZBP1 (de Reuver et al. 2022; Jiao et al. 2022; Zhang et al. 2022).

The p150 isoform has been considered key to suppressing MDA5-mediated dsRNA sensing. This is primarily evidenced by the phenotypes of ADAR1p150-specific knockout mice (*Adar1p150*^−*/*−^), generated by the deletion of the first exon of the gene (Ward et al. 2011c). The *Adar1p150*^−*/*−^ mutant was embryonic lethal at E11.0–E12.0, similar to the *Adar1*^−*/*−^ that lacked both p110 and p150 isoforms (Hartner et al. 2004; Ward et al. 2011c). *Adar1p150*^−*/*−^ embryos had abnormal morphology, and cells derived from them were more susceptible to viral infection (Ward et al. 2011c). Concurrent deletion of MAVS could rescue the embryonic lethality of the *Adar1p150*^−*/*−^ mice, with animals surviving to weaning (Pestal et al. 2015). In contrast, mice without the p110 isoform (*Adar1p110*^−*/*−^) have normal embryonic development but a high post-natal mortality (Kim et al. 2021). The p110-deficient mice had no ISG signature indicating that the retained expression of the p150 isoform was sufficient to prevent MDA5 activation. Deletion of MDA5 in the *Adar1p110*^−*/*−^ model (*Adar1p110*^−*/*−^*Ifih1*^−*/*−^) failed to prevent the high level of postnatal lethality; however, expression of a single editing dead allele of ADAR1 (*Adar1p110*^−*/E861A*^) could rescue viability (Kim et al. 2021). The above studies suggest that ADAR1p150, but not ADAR1p110, is required to suppress MDA5 activation during embryonic development.

Outside of identifying the day of embryonic lethality of the *Adar1p150*^−*/*−^ animals, there was no further in vivo characterization of the original *Adar1p150*^−*/*−^ allele (Ward et al. 2011c). Furthermore, the isoform specificity of the original *Adar1p150*^−*/*−^ allele and whether the method used for gene targeting may affect the expression of the retained ADAR1p110 expression remains an unresolved question (Matthaei et al. 2011; Steinman and Wang 2011; Ward et al. 2011a,b,c). In this study, we have identified and characterized a new *Adar1p150*^−*/*−^ mouse allele. This allele has a single nucleotide deletion which caused a frameshift and in-frame stop codon, leading to the specific loss of ADAR1p150. Critically, this point mutation did not interfere with either the constitutive or IFN-inducible promoters of ADAR1, so the expression of ADAR1p110 remained unaffected. We have characterized the in vivo functions of ADAR1p150 during development and adult homeostasis to allow comparison to the *Adar1* null and editing deficient alleles. The model provides a new tool for future studies delineating the isoform-specific effects of ADAR1 and its role in disease.

## RESULTS AND DISCUSSION

### Identification of a new *Adar1p150*-specific loss-of-function allele

**FIGURE 1.**
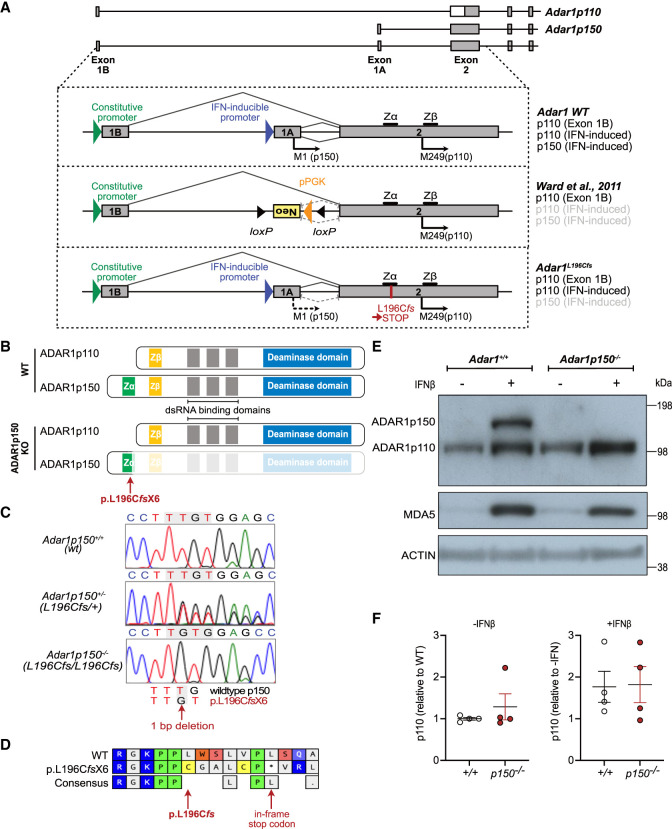
*Adar1*^*L196Cfs*^ mutation leads to an isoform-specific deletion of ADAR1p150 but retention of p110. (*A*) Genomic organization of the murine *Adar* locus showing locations of alternative exon 1A/1B and exon 2 and individual promoters and initiation (M1 and M249) codons that lead to expression of two ADAR1 isoforms. The IFN-inducible promoter upstream of exon 1A leads to the ADAR1p150 expression from M1, and ADAR1p110 from M249 when leaky ribosome scanning occurs. The constitutive promoter upstream of exon 1B maintains the constitutive expression of ADAR1p110. The schematic provides the comparison of the locus and protein products from wild-type (WT) *Adar*, the previous ADAR1p150-deficient model (Ward et al. 2011c) and the L196C*fs* allele described in this report. (*B*) Schematic of the WT ADAR1 isoforms p110 and p150 and the location of the L196C*fs* mutation that leads to knockout of the ADAR1p150 protein. (*C*) Sanger sequencing chromatogram and alignments of genomic DNA isolated from *Adar1*^*+/+*^, *Adar1p150*^+/−^ (*Adar1*^*L196Cfs/+*^), and *Adar1p150*^−*/*−^ (*Adar1*^*L196Cfs/L196Cfs*^) animals indicating 1 bp deletion in the L196C*fs* mutation. (*D*) Predicted amino acid translation of the WT ADAR1p150 and L196C*fs* allele. (*E*) Western blot analysis of WT *Adar1*^*+/+*^ and *Adar1p150*^−*/*−^ mouse embryonic fibroblasts (MEFs) after 24 h of IFN-beta (IFNβ) treatment. Expression of ADAR1 isoforms p150 and p110, MDA5 as well as ACTIN are indicated. (*F*) Quantification of the western blots is shown in (*E*) and Supplemental Figure S1C. ADAR1p110 expression was normalized to ACTIN. The *left* panel shows ADAR1p110 expression at baseline normalized to *Adar1*^*+/+*^. The *right* panel shows ADAR1p110 levels plus IFN normalized to minus IFN for each cell line. *N* = 4 for both *Adar1*^*+/+*^ (+/+) and *Adar1p150*^−*/*−^ (*p150*^−*/*−^) generated from three independent mice. The figure presented here is the replicate of the samples in lanes 3–6 of Supplemental Figure S1C (*left* image) from a different batch of IFNβ treatment. Data represent the mean ± SEM.

The two isoforms of ADAR1 are produced by alternative use of the first exon via different promoters. ADAR1p150 is produced from a type I IFN-inducible promoter that is located upstream of exon 1A (Fig. 1A). The initiation of translation of the p150 isoform starts at M1 within exon 1A and the entire exon 2 functions as a coding sequence for p150. In contrast, the p110 isoform is expressed from a constitutive promoter upstream of exon 1B which is spliced into exon 2, where the p110-specific initiation codon M249 (human M296) is located (George et al. 2005). ADAR1p110 can also be produced from the IFN-inducible promoter due to inefficient translation initiation at M1, leading to the initiation of translation of p110 at M249 in exon 2 (Wong et al. 2003; George et al. 2005; Sun et al. 2021). The IFN-inducible expression of p110 alongside p150 may contribute to optimal editing during the IFN response (Sun et al. 2021). The previously described ADAR1p150 knockout mouse allele was generated by substituting the IFN-inducible promoter and exon 1A with an antisense-orientated PGK-Neo cassette (Fig. 1A; Ward et al. 2011c).

During the generation of a p.P195A knock-in allele using CRISPR/Cas9 (Liang et al. 2023), we identified an incidental mutation that was predicted to result in a p150 isoform-specific knockout allele. A single nucleotide deletion at nucleotide 587 (587delT) was identified (nomenclature based on NCBI CCDS50963). This was predicted to result in a leucine 196 to cysteine (p.L196C*fs*X6) amino acid change and introduction of in-frame stop codon(s) due to the change in the reading frame (Fig. 1B–D). We tested whether the L196C*fs* mutant sequence would give rise to a protein product. We transduced HEK293T cells with a lentivirus expressing an amino-terminal NeonGreen fusion protein with either ADAR1p150 WT or a cDNA encoding the ADAR1^L196C*fs*^ mutation. In the p150 WT cells, NeonGreen was apparent in the cytoplasm as expected (Supplemental Fig. S1A, top panels). In the L196C*fs* expressing cells, no or a very low level of NeonGreen was detectable (Supplemental Fig. S1A, bottom panels). Protein lysates from the HEK293T cells were probed with an anti-NeonGreen antibody. In cells expressing NeonGreen-ADAR1p150 WT, a fusion protein size of ∼178 kDa was detected by either the anti-NeonGreen or anti-ADAR1 antibody indicating the expression of NeonGreen and full-length ADAR1p150 protein (Supplemental Fig. S1B). In NeonGreen-ADAR1^L196C*fs*^ cells, a very low to barely detectable level of a ∼50 kDa fusion protein was detected with the NeonGreen antibody. Given that this small amount of protein was produced under optimal expression conditions with a strong viral promoter, it is likely the protein produced at the native locus is negligible. Any truncated protein from the L196C*fs* allele would contain a partial Zα domain lacking the critical residues for Z-RNA-binding such as W197; however, the L196 itself is not involved in the binding. For a detailed description of the key residues of the Zα domain, see the review paper by Nakahama and Kawahara (2021). We therefore conclude that the L196C*fs* mutation resulted in a null *Adar1p150* allele.

We confirmed the introduction of the mutation in the germ-line by Sanger sequencing and then inbred heterozygous mice to assess this new allele. We generated and immortalized MEFs from E11.5 embryos and treated them with murine IFN-beta (IFNβ) to assess ADAR1p150 expression. The *Adar1*^*+/+*^ MEFs expressed ADAR1p110 basally, and ADAR1p150 was robustly induced upon treatment with IFNβ. An elevation of ADAR1p110 protein was observed following IFNβ treatment of *Adar1*^*+/+*^ MEFs (Fig. 1E). Homozygous *Adar1*^*L196Cfs/L196Cfs*^ (referred to herein as *Adar1p150*^−*/*−^) MEFs had comparable levels of ADAR1p110 to WT cells at baseline and had no expression of ADAR1p150 following IFNβ treatment. Importantly, the expression of ADAR1p110 was equivalent in *Adar1p150*^−*/*−^ versus *Adar1*^*+/+*^ MEFs, both at baseline and following induction by IFNβ treatment, indicating ADAR1p110 expression and induction was unaffected by the point mutation (Fig. 1F; Supplemental Fig. S1C). Therefore, the *Adar1*^*L196Cfs*^ point mutation generated a *Adar1p150*-null allele without any apparent effect on ADAR1p110 expression.

Our *Adar1p150*-specific knockout contrasts with the previous model, which used an inverted PGK-Neo cassette to disrupt the p150 isoform (Fig. 1A). Analysis of the available published western blots from the existing *Adar1p150*^−*/*−^ model indicates that the retained ADAR1p110 may not be expressed comparably to the *Adar1p150*^+/−^ sample, nor is it induced by type I IFN treatment in a manner similar to the control used in those studies (Ward et al. 2011b; Pestal et al. 2015). Therefore, two potential caveats of the originally developed *Adar1p150*^−*/*−^ allele are (1) the IFN-inducible expression of ADAR1p110 may be reduced due to the disruption of the IFN promoter, and (2) the inverted PGK-Neo cassette may impact ADAR1p110 expression from both the targeted and nontargeted allele via transcriptional interference and/or production of aberrant antisense RNAs, as has been reported for other alleles using this method of gene disruption (Scacheri et al. 2001; Matthaei et al. 2011; Steinman and Wang 2011; Ward et al. 2011c). This may be particularly relevant when the allele is used to study in vivo function of p150 and p110 isoforms or when paired with other mutants as a compound heterozygote (Pestal et al. 2015; Maurano et al. 2021; Hubbard et al. 2022).

### ADAR1p150-specific knockout mice are embryonic lethal

**FIGURE 2.**
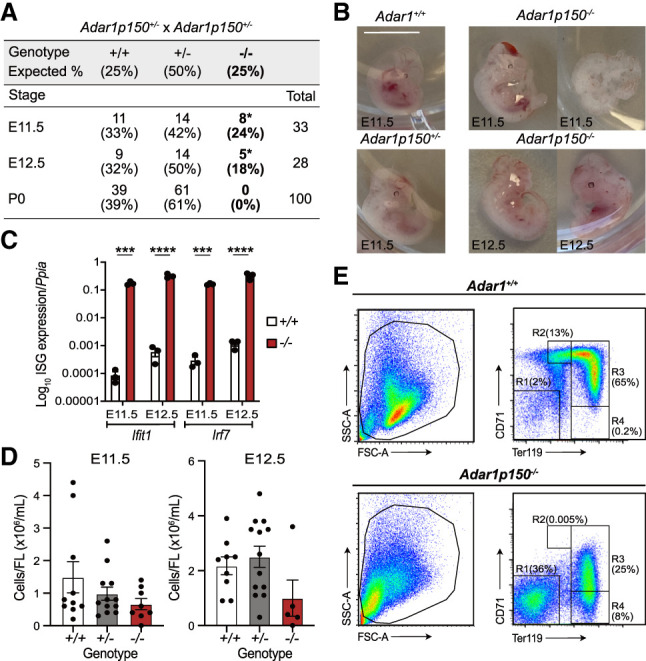
ADAR1p150-specific knockout mice die mid-gestation at E11.5–E12.5. (*A*) Survival and frequency of *Adar1*^+/+^, *Adar1p150*^+/−^, and *Adar1p150*^−*/*−^ mice at three development stages E11.5, E12.5, and P0 (day of birth) from *Adar1p150*^+/−^ inter-crosses. (*B*) Representative images of embryos of the indicated genotypes at E11.5 or E12.5. The scale bar represents 0.5 cm. (*C*) Expression of *Ifit1* and *Irf7*, both ISGs, in *Adar1*^+/+^ and *Adar1p150*^−*/*−^ embryos’ at E11.5 and E12.5. Data expressed as log_10_ gene expression relative to *Ppia* expression (reference gene). Three independent samples were used for each indicated genotype and developmental stage. Significance was determined by a two-way ANOVA test with Bonferroni's multiple comparisons with statistical significance of (***) *P* < 0.001 and (****) *P* < 0.0001. Data represent the mean ± SEM. (*D*) Cellularity of FL from E11.5 (*left*) and E12.5 (*right*). At E11.5, the number of *Adar1*^+/+^ (+/+) *n* = 10, *Adar1p150*^+/−^ (+/−) *n* = 12, and *Adar1p150*^−*/*−^ (−/−) *n* = 8. At E12.5, the number of +/+ *n* = 9, +/− *n* = 13, and −/− *n* = 5. Significance was determined by a one-way ANOVA test with Dunnett's multiple comparisons. Error bars are SEM. (*E*) Representative flow cytometry profiles of FL erythroid cells of interest at E12.5 with SSC-A/FSC-A (*left*); the proportion of erythroblast population of the viable FL erythroid cells labeled with CD71/Ter119 (*right*). R1 = CD71^−^Ter119^−^, R2 = CD71^high^Ter119^med^, R3 = CD71^high^Ter119^high^, and R4 = CD71^−^Ter119^high^.

Having established that our allele was a *Adar1p150*-null allele, without impacting ADAR1p110 expression, we sought to characterize its phenotype. *Adar1p150*^+/−^ heterozygous mice were fertile and had no discernible phenotype. We bred *Adar1p150*^+/−^ animals to determine the survival of *Adar1p150*^−*/*−^ mice. No live *Adar1p150*^−*/*−^ pups were identified (Fig. 2A). Based on the embryonic lethality of the *Adar1*^−*/*−^ mice (Hartner et al. 2004, 2009; Wang et al. 2004), we assessed embryos at E11.5 and E12.5 and found *Adar1*^*+/+*^, *Adar1p150*^+/−^, and *Adar1p150*^−*/*−^ embryos were present at the expected Mendelian ratio (Fig. 2A). *Adar1p150*^−*/*−^ embryos, while present, showed evidence of developmental abnormalities and reduced viability between E11.5 and E12.5. At E11.5, three of the eight *Adar1p150*^−*/*−^ embryos had a normal appearance whereas the other five had morphological abnormalities. At E12.5, all *Adar1p150*^−*/*−^ embryos had abnormal features. The *Adar1p150*^−*/*−^ embryos were fragile and had a pale color. Some abnormal embryos showed hemorrhage on the back or the head (Fig. 2B). Similar features were previously reported for the *Adar1*^−*/*−^ mutant, lacking both p110 and p150, with embryonic lethality at E11.0–E12.5 (Hartner et al. 2004, 2009; Wang et al. 2004). Given the importance of ADAR1 in inhibiting the activation of MDA5 and downstream ISGs, we performed qRT-PCR on tissue from the embryos. At both E11.5 and E12.5, *Adar1p150*^−*/*−^ animals had a profound induction of the ISGs *Ifit1* and *Irf7* (Fig. 2C) compared to the littermate WT controls. The expression of *Ifit1* and *Irf7* was higher at E12.5 than in E11.5 in the *Adar1p150*^−*/*−^ embryos (Supplemental Fig. S1D). The level of ISGs in the previous *Adar1p150*^−*/*−^ mutant was not reported. The embryonic lethality of our *Adar1p150*^−*/*−^ is consistent with the originally described *Adar1p150*^−*/*−^ allele, which died between E11.0 and E12.0 (Ward et al. 2011c).

Detailed phenotypic analysis of *Adar1p150*^−*/*−^ embryos has not been reported. Previous studies found that both *Adar1* null (*Adar1*^−*/*−^) and the editing deficient (*Adar1*^*E861A/E861A*^) embryos had a failure in hematopoiesis in FL (Hartner et al. 2004, 2009; Wang et al. 2004; Liddicoat et al. 2015). The FL is the primary site of developmental hematopoiesis, with erythropoiesis (red cell production) being the most important lineage during early development. ADAR1 protein-deficient erythroid cells had an increased rate of cell death and there was a loss of erythroid progenitors indicating ADAR1 is essential for normal erythropoiesis (Hartner et al. 2004, 2009; Liddicoat et al. 2016). The specific requirement of ADAR1p150 in hematopoiesis during embryonic development had not been reported. *Adar1p150*^−*/*−^ FL had fewer cells at both E11.5 and E12.5, although there was no statistical difference (Fig. 2D). In one of the *Adar1p150*^−*/*−^ FLs with enough viable cells for flow cytometry analysis, the p150-deficient FL had a reduction of viable erythroid cells (Fig. 2E, left). The expression of CD71/Ter119 markers identifies the differentiation stage of erythroid cells, with cells progressing from the R1 to R4 populations with maturation (Socolovsky et al. 2001; Koulnis et al. 2011). The *Adar1p150*^−*/*−^ embryos had increased R1 with defective maturation including an absence of R2 and severely reduced R3 (Fig. 2E, right), indicating defective erythropoiesis in the FL in the absence of ADAR1p150.

These data demonstrate that the ADAR1p150 isoform is essential for embryonic development in vivo. The embryonic lethality, abnormal embryo appearance, abnormal FL hematopoiesis, and high ISG signature of the *Adar1p150*^−*/*−^ embryos reproduced the previously described phenotypes observed in both *Adar1*^−*/*−^ or *Adar1*^*E861A/E861A*^ mutants (Hartner et al. 2004, 2009; Wang et al. 2004; Liddicoat et al. 2015). Taken together, the essential physiological function of ADAR1 in fetal hematopoiesis and suppressing IFN signaling are mediated by ADAR1p150. The retained expression of ADAR1p110 cannot compensate for this function.

### ADAR1p150 is essential for adult homeostasis and hematopoiesis in vivo

**FIGURE 3.**
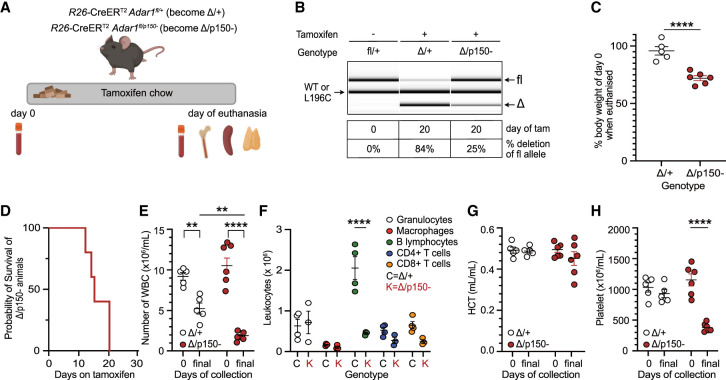
Somatic deletion of ADAR1p150 in adult mice is lethal. (*A*) Illustration of somatic deletion model. Upon tamoxifen treatment, *R26*-CreER *Adar1*^*fl/+*^ and *R26*-CreER *Adar1*^*fl/p150*−^ animals became Δ/+ and Δ*/p150*−, respectively. (*B*) Representative genotyping of genomic DNA and percentage deletion of the floxed allele on the day of euthanasia using DNA isolated from whole BM. Image and recombination percentages were calculated using LabChip (PerkinElmer). (*C*) Percentage change in body weight on the day of euthanasia compared to day 0 (before the start of the tamoxifen diet). Δ*/+ (n* = 5) and Δ*/p150*− (*n* = 6). (*D*) Kaplan–Meier survival plot of the Δ*/p150*− animals (*n* = 6). Note the control animals are not plotted and were healthy; each Δ*/p150*− was collected with a control animal on the same day to allow paired analysis. (*E*) Total white blood cell counts (WBC) in peripheral blood (PB) at day 0 (pre-tamoxifen) and day of euthanasia (final). (*F*) Absolute numbers of each lineage in PB on the day of euthanasia (final). (*G*) Hematocrit (HCT) populations in PB at day 0 (pre-tamoxifen) and day of euthanasia (final). (*H*) Total platelets in the PB at day 0 (pre-tamoxifen) and day of euthanasia (final). Statistical comparison in all plots was done by unpaired *t*-test (*C*), two-way ANOVA (*E*,*G*,*H*) with Bonferroni's multiple comparisons or ordinary two-way ANOVA (*F*) tests with statistical significance of (*) *P* < 0.05, (**) *P* < 0.01, (***) *P* < 0.001, and (****) *P* < 0.0001. Data represented as mean ± SEM.

To understand the specific requirement for ADAR1p150 in adult homeostasis, we used an inducible model that we have previously used to assess the somatic restricted expression of *Adar1* null, editing deficient ADAR1 or an AGS-mimicking mutation (Liddicoat et al. 2015; Heraud-Farlow et al. 2017; Liang et al. 2023). We crossed the *Adar1p150*^+/−^ animals to *R26*-CreER^T2^ *Adar1*^*fl/fl*^ mice. This enabled tamoxifen-induced deletion of the floxed *Adar1* allele, leaving mice either heterozygous and retaining expression of p110 and p150 isoforms (*fl*/+ becomes Δ/+) or p150-deficient and only expressing p110 (*fl/p150*− become Δ*/p150*−). *R26*-CreER *Adar1*^*fl*/+^ (control) and *R26*-CreER *Adar1*^*fl/p150*−^ adult mice aged 8–10 wk were fed tamoxifen-containing food for up to 28 d (Fig. 3A). This bypassed the developmental requirement for ADAR1p150. The *Adar1* floxed allele is deleted broadly across cell types and organs upon tamoxifen treatment. We confirmed the efficient recombination of the floxed allele in the bone marrow (BM) of the Δ/+ mice (Fig. 3B; Supplemental Fig. S2A). In contrast, despite being moribund the Δ*/p150*− mice retained a high percentage of the *Adar1* floxed allele, indicating most likely selection against cells that had deleted the floxed allele. This phenomenon of retention of an unexcised allele has previously been seen using the same experimental model with both a *fl/fl* (loss of *Adar1* in both alleles upon tamoxifen) and *fl/E861A* (expressing editing dead ADAR1 upon tamoxifen) genotypes (Liddicoat et al. 2015; Heraud-Farlow et al. 2017).

The loss of ADAR1p150 led to significant weight loss compared to day 0 weights, resulting in euthanasia prior to day 28 due to meeting of ethical endpoints (Fig. 3C). Each moribund Δ*/p150*− animal was assessed in parallel with a paired control Δ/+ on the same day (Fig. 3D; note that the Δ/+ are not represented on the plot and have survived to 28 d of tamoxifen feeding without requiring euthanasia in all prior experiments). Given the characterized impact of ADAR1 loss on hematopoiesis (Hartner et al. 2004, 2009; Liddicoat et al. 2016; Liang et al. 2023), we quantified PB indices of mice on day 0 and on the day of collection (Fig. 3E–H; Supplemental Fig. S2B–F). Reduced WBC counts have been observed following tamoxifen treatment in *R26*-CreER^T2^ mice previously (Smeets et al. 2018); the loss of ADAR1p150 in the Δ*/p150*− mice resulted in a more significant decline of WBC compared to the controls (Fig. 3E). The Δ*/p150*− samples also had reductions in B lymphocyte numbers (Fig. 3F), platelets (Fig. 3H), and mean corpuscular volume (Supplemental Fig. S2D).

**FIGURE 4.**
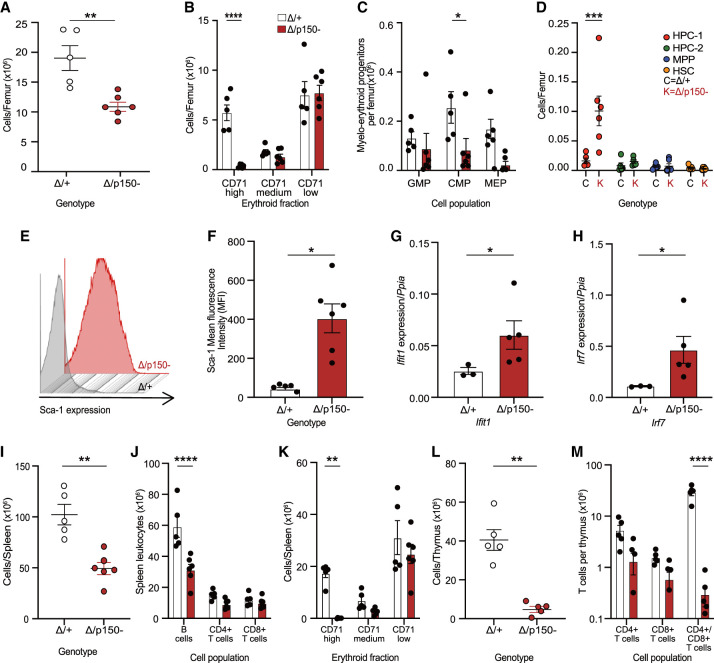
Acute loss of ADAR1p150 disrupted hematopoietic homeostasis in adult mice accompanied by an elevated innate immune response. Analysis of BM, spleen and thymus from *R26*-CreER *Adar1*^Δ/+^ and *R26*-CreER *Adar1*^Δ*/p150*−^ animals described in Figure 3. (*A*) Total cellularity per femur. (*B*) Erythroid cells per femur. (*C*) Numbers of myelo-erythroid progenitors per femur. Cell populations measured were granulocyte–monocyte progenitor (GMP), common myeloid progenitor (CMP), and megakaryocyte–erythrocyte progenitor (MEP) cells. (*D*) The absolute number of stem cell and multipotent progenitor (MPP) populations per femur. Cell populations measured were hematopoietic progenitor cells (HPC) fractions 1 and 2, MPP, and hematopoietic stem cells (HSCs). (*E*) Representative flow cytometry histograms of Sca-1 expression on the lineage negative (lin^−^c-kit^+^) BM fraction. (*F*) Quantification of the mean Sca-1 fluorescence intensity by flow cytometry. (*G*) Normalized expression of *Ifit1* transcripts measured by qRT-PCR in the BM; Δ/+ (*n* = 3) and Δ*/p150*− (*n* = 5). Data expressed as mean ± SEM gene expression relative to *Ppia* expression. (*H*) Normalized expression of *Irf7* transcripts measured by qRT-PCR in the BM; Δ/+ (*n* = 3) and Δ*/p150*− (*n* = 5). Data expressed as mean ± SEM gene expression relative to *Ppia* expression. (*I*) Total viable cellularity of the spleen. (*J*) The absolute number of B lymphocytes, CD4 positive (CD4+), and CD8 positive (CD8+) T lymphocytes in the spleen. (*K*) Erythroid cells in the spleen. (*L*) Total viable cellularity of the thymus. (*M*) The absolute number of CD4 positive (CD4+), CD8 positive (CD8+), and CD4 and CD8 double positive (CD4+/CD8+) T cells in the thymus. Statistical tests used were unpaired *t*-test (*A*,*F*,*G*,*H*,*I*,*L*) and two-way ANOVA (*B*,*C*,*D*,*J*,*K*,*M*) with Bonferroni's multiple comparisons with statistical significance of (*) *P* < 0.05, (**) *P* < 0.01, (***) *P* < 0.001, and (****) *P* < 0.0001. Data expressed as mean ± SEM. In (*A*–*F*) and (*I*–*M*), Δ*/+ (n* = 5) and Δ*/p150* − (*n* = 6).

We further assessed the requirement for ADAR1p150 in hematopoiesis by collecting and analyzing the BM, spleen, and thymus on the day of euthanasia (Fig. 4; Supplemental Fig. S2G–I). Previous work demonstrated the requirement of ADAR1 in the maintenance of adult hematopoiesis in mice (Hartner et al. 2009; Heraud-Farlow et al. 2017; Nakahama et al. 2018). In these prior studies, it was determined that acute deletion of ADAR1 resulted in hematopoietic failure because of both cell intrinsic requirements in mature lineages and for sustaining hematopoietic output from hematopoietic stem and progenitor populations in the BM (Hartner et al. 2009). In the BM, there was a reduction in the total cellularity (Fig. 4A) and a significant loss of CD71+/Ter119+ erythroid cells (Fig. 4B). Common myeloid progenitors are generated from HSCs and can further differentiate into either GMP, or MEP cells (Akashi et al. 2000). The number of CMPs were significantly decreased by the acute loss of ADAR1p150, indicative of an impairment in adult myeloid progenitors (Fig. 4C). The HPC, MPP, and HSCs can be identified in the Lineage^−^Sca-1^+^c-Kit^+^ (LSK+) fraction of BM cells using CD150 and CD48 markers (Oguro et al. 2013; Pietras et al. 2015). The population of HPC was abnormal in the Δ*/p150*− mice, while the MPP and HSCs were comparable with the control mice (Fig. 4D).

Loss of ADAR1 or A-to-I editing by ADAR1 led to a profound increase in the ISG expression (Hartner et al. 2009; Liddicoat et al. 2015). The activation of the innate immune/interferon pathway in Δ/+ and Δ*/p150*− was first assessed by measuring the cell surface expression of Sca-1, an IFN-induced cell surface protein, by flow cytometry. There was a ∼10-fold up-regulation of Sca-1 expression on the BM cells of p150-deficient animals (Fig. 4E,F). The expression of two hallmark ISGs, *Ifit1* and *Irf7*, was assessed by qRT-PCR in BM cells. There was an elevated expression of both genes in the Δ*/p150*− BM (Fig. 4G,H).

In the spleen, there was a reduced weight and cellularity (Fig. 4I; Supplemental Fig. S2H). This was due to reduced numbers of B lymphocytes (Fig. 4J) and erythroid cells (Fig. 4K). The thymus had reduced cellularity and weight (Fig. 4L; Supplemental Fig. S2I). Consistent with previous work, the number of CD4+/CD8+ T cells was drastically reduced suggesting the importance of ADAR1p150 in thymic T cell maturation (Fig. 4M; Nakahama et al. 2018). Taken together, all results demonstrate a continuous requirement for ADAR1p150 for maintenance of normal hematopoiesis in adult mice, consistent with a generalized failure in hematopoiesis as seen in germ-line *Adar1* null, editing dead, and cell-specific conditional *Adar1* knockout models (Hartner et al. 2009; Liddicoat et al. 2015, 2016; Heraud-Farlow et al. 2017; Nakahama et al. 2018).

The new ADAR1p150-specific knockout mouse model described here has allowed a more detailed characterization of the in vivo functions of ADAR1p150. It also provides a tool to address long-standing questions about the role of ADAR1p150 in suppressing different dsRNA sensors such as MDA5, PKR, and RNaseL. Abundant in vivo work has demonstrated MDA5-mediated immune activation in the absence of ADAR1 in both human cells and mice (Mannion et al. 2014; Liddicoat et al. 2015; Pestal et al. 2015; Heraud-Farlow et al. 2017). Studies in various cell lines and mouse models have also demonstrated a role for ADAR1 in the suppression of PKR (Wang et al. 2004; Li et al. 2010; Chung et al. 2018; Gannon et al. 2018; Maurano et al. 2021), OAS-RNaseL (Li et al. 2017), and ZBP1 (de Reuver et al. 2022; Hubbard et al. 2022; Jiao et al. 2022; Zhang et al. 2022). These sensors exert direct antiviral activities following dsRNA sensing through translational shutdown, RNA cleavage or triggering cell death pathways, respectively (Levin and London 1978; Zhou et al. 1993; Upton et al. 2019; Zhang et al. 2020). To assess PKR activation, we treated *Adar1p150*^−*/*−^ or control MEFs with IFNβ for 24 h and performed immunoblotting (Supplemental Fig. S3A). Sensing of dsRNA by the kinase PKR triggers phosphorylation of eIF2α and translational shutdown (Levin and London 1978). As there is no specific antibody for p-PKR in mouse, p-eIF2α is used as a proxy for PKR activation. Following IFNβ treatment of MEFs there was a higher induction of p-eIF2α in *Adar1p150*^−*/*−^ MEFs compared to the control suggesting ADAR1p150 protein may suppress IFNβ-induced PKR activation. We do not see evidence for RNA degradation and activation of OAS-RNaseL in brain, liver, or heart tissue from *Adar1*^−*/*−^*Ifh1*^−*/*−^ mice and therefore did not test this in the *Adar1p150*^−*/*−^ mice (Supplemental Fig. S3B). In a parallel study, we have directly assessed the role of dsRNA sensors where we interbred *Adar1p150*^+/−^ mice with *Ifih1*^−*/*−^ (MDA5 KO) and *Eif2ak2*^−*/*−^ (PKR KO) mice (Hu et al. 2023). Knockout of MDA5 allowed *Adar1p150*^−*/*−^ mice to survive to shortly after birth, similar to the rescue of fully *Adar1*^−*/*−^ animals. Concurrent deletion of MDA5 and PKR generated a full Mendelian rescue of *Adar1p150*^−*/*−^, with *Adar1p150*^−*/*−^*Ifih1*^−*/*−^*Eif2ak2*^−*/*−^ animals surviving to adulthood with apparent normal development and lifespan. This demonstrated that MDA5 and PKR are the primary effectors of lethal autoimmune activation that led to the death of *Adar1p150*^−*/*−^ mice.

In this work, we characterized a new *Adar1p150*^−*/*−^ and demonstrated that this allele resulted in the specific loss of the ADAR1p150 isoform, without any apparent impacts on the expression of the p110 isoform, both basally and following IFNβ treatment. We completed analysis that allowed a direct comparison to previous studies describing the phenotypes of *Adar1* null (loss of both p110 and p150), editing deficient mutants (E861A), and AGS-mimicking mutant (P195A) (Hartner et al. 2004, 2009; Wang et al. 2004; Liddicoat et al. 2015; Liang et al. 2023). This provides an important comparison of these distinct alleles. When considered together with the recently described *Adar1p110*^−*/*−^ allele, we conclude that ADAR1p150 is specifically required to suppress the activation of the innate immune response and allow normal development and adult homeostasis. This is the physiologically essential and nonredundant function of ADAR1p150.

## MATERIALS AND METHODS

### Ethics statement

All animal experiments were approved by the Animal Ethics Committee of St. Vincent's Hospital, Melbourne, Australia (Protocol number 016/20). Animals were euthanized by CO_2_ asphyxiation or cervical dislocation.

### Animals

*Adar1*^*L196Cfs*^ mice were identified as an incidental mutation arising from CRISPR/Cas9 targeting in C57BL/6 zygotes to generate a p.P195A knock-in point mutation by the Monash Genome Modification Platform (Monash University) (Liang et al. 2023). A single nucleotide deletion at nucleotide 587 (587delT) resulted in the p.L196C*fs*X6 mutation (nomenclature based on NCBI CCDS50963). The introduction of the mutation was confirmed by Sanger sequencing of the region in both the founders and subsequent generations. *Adar*^*fl/fl*^ (*Adar1*^*fl/fl*^; exon 7–9 floxed; MGI allele: *Adar*^*tm1.1Phs*^; MGI:3828307) (Hartner et al. 2004, 2009) and *Rosa26*-CreER^T2^ (Gt(ROSA)26Sor^tm1(cre/ERT2)Tyj^) (Ventura et al. 2007) mice were on a backcrossed C57BL/6 background as previously described (Hartner et al. 2009; Liddicoat et al. 2015, 2016; Heraud-Farlow et al. 2017). All animals were housed at the BioResource's Centre at St. Vincent's Hospital, Melbourne, Australia. Mice were maintained and bred under specific pathogen-free conditions with food and water provided ad libitum. For the somatic deletion models *R26*-CreER *Adar1*^*fl/+*^ and *R26*-CreER *Adar1*^*fl/p150*^, all animals were aged from 8 to 10 wk at the initiation of tamoxifen; tamoxifen-containing food was prepared at 400 mg/kg tamoxifen citrate (Selleckchem) in standard mouse chow (Specialty Feeds).

### Genotyping

Genotyping of the L196C mutants was determined by PCR and Sanger sequencing. The following primers: primer P1 (5′-ACCATGGAGAGGTGCTGACG-3′) and P2 (5′-ACATCTCGGGCCTTGGTGAG-3′), were used to obtain a 489 bp product. The purified PCR product was sequenced using P1 primer (Australian Genome Research Facility). Genotyping of all other lines and Cre recombination was performed as previously described by Liddicoat et al. (2015) and Heraud-Farlow et al. (2017).

### Embryo and fetal liver analysis

Timed mating of *Adar1p150*^+/−^ females was undertaken for embryo analysis, and embryos were collected at E11.5 and E12.5. Fetal livers (FL) were isolated from the embryos and suspended in 1 mL of PBS containing 2% FBS using a 21G needle/1 mL syringe. FL cell counts were performed on a hematological analyzer (Sysmex KX1000). Single-cell FL suspensions were then subjected to antibody staining for flow cytometry analysis. The measurement of erythroid cells used antibodies against murine Ter119 (PE), CD71 (APC), and CD44 (PE-Cy7) sourced from eBioscience, BioLegend, or BD Pharmingen. Cells were acquired on a BD LSRII Fortessa and analyzed with FlowJo software version 9 or 10.0 (TreeStar).

### Mouse embryonic fibroblasts and immortalization

Mouse embryonic fibroblasts (MEFs) were generated from E11.5 embryos of the indicated genotypes. The embryos were dissected, the head was used for qRT-PCR, and the heart and FL (for flow cytometry) were removed. MEFs were made from the remaining embryo. The tissue was drawn through an 18G needle/1 mL syringe, suspended in 1 mL of 0.025% trypsin-EDTA (Gibco/Thermo Fisher), and incubated at 37°C in a 10 cm^2^ tissue culture plate for 30 min. Then, 10 mL of normal growth media (High glucose DMEM [Sigma] containing 10% FBS [not heat-inactivated, Assay Matrix], 1% penicillin/streptomycin [Gibco], 1% glutamax [Gibco], and 1% amphotericin B [Sigma; 250 µg/mL stock]) was directly added to the plate. The digested tissue was resuspended and dispersed. The MEFs were incubated in a hypoxia chamber flushed with 5% oxygen and 5% carbon dioxide in nitrogen at 37°C. Once the cells were confluent (>70% confluency), the cells were trypsinized and passaged onto 10 cm^2^ plates in normoxic conditions for all further culturing. MEFs were immortalized with 1 mL of media containing an shRNA targeting murine p53 (shp53.1224 in LMP vector), 1% polybrene, and normal growth media (Dickins et al. 2005). After 12 h of infection, 1 mL of fresh media was added to the cells. After 72 h, the media was replaced with fresh normal growth media. The immortalized MEFs were treated with recombinant murine interferon beta (PBL Assay Science; PBL-12405) at 250 U/mL for 24 h in normal growth media.

### Western blot analysis

MEFs or HEK293T cells were collected by trypsinization, and pellets washed in cold PBS and resuspended in RIPA buffer (20 mM Tris-HCl, pH 8.0, 150 mM NaCl, 1 mM EDTA, 1% sodium deoxycholate, 1% Triton X-100, and 0.1% SDS) supplemented with 1× HALT Protease Inhibitor and 1× PhosSTOP Phosphatase Inhibitor (Thermo Fisher Scientific). Lysates were used for western blot analysis as described below. Protein was quantified using the Pierce BCA Protein Assay Kit (Thermo Fisher Scientific) on an Enspire multimode plate reader (PerkinElmer). Ten or 20 µg of protein extract per sample and protein markers (SeeBlue Plus2 Pre-stained Protein Standard [Invitrogen]) were loaded on precast NuPAGE 10% or 4%–12%, Bis-Tris polyacrylamide gels (Invitrogen) and transferred onto Immobilon-P PVDF membranes (Merck Millipore). Membranes were blocked with 5% milk in Tris-buffered saline with tween (TBST) and incubated at 4°C overnight with rat monoclonal anti-mouse ADAR1 antibody (clone RD4B11) (Liddicoat et al. 2015), rabbit anti-MDA-5 (Cell Signaling, D74E4), rabbit anti-PKR (Abcam, EPR19374), phospho-eIF2α (anti-EIF2S1 [phospho S51], Abcam, ab32157), total eIF2α (Cell Signaling Technology, 5324), mouse anti-NeonGreen (Chromotek 32F6), and mouse anti-ACTIN (Sigma Aldrich, A1978). Membranes were then probed with HRP-conjugated goat anti-rat (Thermo Fisher Scientific, 31470), anti-rabbit (Thermo Fisher Scientific, 31460), or anti-mouse (Thermo Fisher Scientific, 31444) secondary antibodies and visualized using ECL Prime Reagent for chemiluminescent detection on Hyperfilm ECL (Amersham) or the iBright FL1500 Imaging system (Thermo Fisher). Western band intensities were quantified using Fiji.

### Peripheral blood analysis

Peripheral blood was obtained via retro-orbital bleeding into BD Microtainer K2E tubes (Becton Dickinson) from the somatic mutation models. PB samples were counted on a hematological analyzer (Sysmex KX1000). The red blood cells were lysed using hypotonic lysis buffer (150 mM NH_4_Cl, 10 mM KHCO_3_, 0.1 mM Na_2_EDTA, pH 7.3) and resuspended in 50 µL of FACS buffer for flow cytometry analysis.

### Flow cytometry analysis

Antibodies against murine B220 (APC-eFluor780), CD11b/Mac-1 (PE), Gr1 (PE-Cy7), F4/80 (APC), CD4 (eFluor450) and CD8a (PerCP-Cy5.5), Ter119 (PE), CD71 (APC), CD44 (PE-Cy7), Sca-1(PerCP-Cy5.5), c-Kit (APC-eFluor780), CD150 (PE), CD48 (PE-Cy7), CD34(eFluor660), CD16/32 (eFluor450), and biotinylated antibodies (CD2, CD3e, CD4, CD5, CD8a, B220, Gr-1, CD11b/Mac1) were used. The biotinylated antibodies were detected with streptavidin-conjugated Brilliant Violet 786. All antibodies were obtained from eBioscience, BioLegend, or BD Pharmingen (Singbrant et al. 2011; Smeets et al. 2014; Liddicoat et al. 2015; Heraud-Farlow et al. 2017). Cells were acquired on a BD LSRII Fortessa and analyzed with FlowJo software version 9 or 10.0 (TreeStar).

### qRT-PCR

Whole heads from embryos or single-cell suspensions of BM from the somatic deletion models were collected and immediately snap frozen in liquid nitrogen or dry ice and stored at −80°C. Frozen tissues were homogenized in TRIsure reagent (Bioline) using IKA T 10 Basic S5 Ultra-Turrax Disperser. RNA was isolated using Direct-Zol columns (Zymo Research) following the manufacturer's instructions and cleaned up with Zymo Clean & Concentrator Kit. Complementary DNA (cDNA) was synthesized using the Tetro cDNA Synthesis Kit (Bioline). qRT-PCR of ISGs (*Ifit1* and *Irf7*) was performed using SYBR green and the ΔΔCT method (normalized to *Ppia*) as previously described (Heraud-Farlow et al. 2017). Duplicate or triplicate reactions per sample were measured using an AriaMx Real-time PCR machine (Agilent). The following primers were used: *Ifit1* P1 (5′-ATGGGAGAGAATGCTGATGG-3′); *Ifit1* P2 (5′-AGGAACTGGACCTGCTCTGA-3′); *Irf7* P1 (5′-CCAGTTGATCCGCATAAGGT-3′); *Irf7* P2 (5′-AGCATTGCTGAGGCTCACTT-3′); *Ppia* P1 (5′-GTCAACCCCACCGTGTTCTT-3′); and *Ppia* P2 (5′-CTGCTGTCTTTGGAACTTTG-3′).

### Plasmid generation and lentivirus production/infection

We generated cDNAs using gBlocks (IDT DNA) to generate an amino-terminal fusion of NeonGreen to an existing *Adar1p150* cDNA (GeneArt) (Mendez Ruiz et al. 2023). The NeonGreen coding sequence was placed upstream of the WT and 587delT (encoding the frameshift and L196C*fs* allele) p150 sequence. Final plasmids were verified by Sanger sequencing. The cDNAs were then subcloned into pLVX (Clontech). To produce lentivirus, HEK293T cells were plated in medium (high glucose DMEM [Sigma] containing 10% FBS, 1% penicillin/streptomycin [Gibco], and 1% glutamax [Gibco]) on 10 cm tissue culture plates and cotransfected with 10 µg of the pLVX-Adar1 vectors, 6.5 µg psPAX2, and 3.5 µg VSVg. psPAX2 was a gift from Didier Trono (Addgene plasmid # 12260; http://n2t.net/addgene:12260; RRID:Addgene_12260). At 48 and 72 h after transfection, viral-containing supernatant was collected. HEK293T cells were infected with 48 h of lentivirus expressing NeonGreen-ADAR1p150 WT and NeonGreen-ADAR1L196C*fs*. Infected cells were selected by puromycin (1 µg/mL) for 3 d and expanded for further analysis. Microscopy images were captured by Olympus IX-81 inverted fluorescence microscope using a 20× objective lens.

### Statistical analysis and figure preparation

To determine statistical significance, unpaired *t*-tests and one-way or two-way ANOVA tests with Dunnett's or Bonferroni's multiple comparisons were conducted in GraphPad Prism software version 9 (GraphPad). Throughout this study, significance is indicated using the following convention: *P < 0.05; **P < 0.01; ***P < 0.001; and ****P < 0.0001. Data are presented as mean ± SEM. The number of samples used is described in the corresponding ﬁgure legends. The figures were generated using BioRender.com and Affinity Designer.

## SUPPLEMENTAL MATERIAL

Supplemental material is available for this article.

## Figures and Tables

**Zhen Liang:**
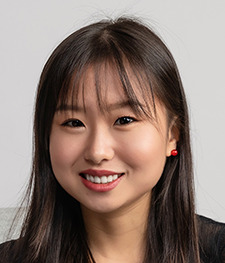


## MEET THE FIRST AUTHOR

***Meet the First Author(s)* is an editorial feature within *RNA*, in which the first author(s) of research-based papers in each issue have the opportunity to introduce themselves and their work to readers of *RNA* and the RNA research community. Zhen Liang is the first author of this paper, “Generation of a new *Adar1p150*^*−/−*^ mouse demonstrates isoform-specific roles in embryonic development and adult homeostasis.” Zhen is a PhD student at the Cancer and RNA Biology laboratory of St. Vincent's Institute of Medical Research and the University of Melbourne. Her current research focuses on how mutations in an RNA editing enzyme ADAR1 can lead to the development of disease and the physiological requirements of ADAR1 in normal development.**

**What are the major results described in your paper and how do they impact this branch of the field?**

The major results described in our paper highlight the crucial role of the cytoplasmic isoform of the RNA editing enzyme adenosine deaminase acting on RNA 1 (ADAR1) in regulating the innate immune response. We characterized a specific knockout mouse model lacking the ADAR1p150 isoform of ADAR1 without compromising the expression of nuclear expressed ADAR1p110. The germline loss of ADAR1p150 led to cell death in the fetal liver, accompanied by an activated IFN response, resulting in embryonic death at E11.5–E12.5. Additionally, somatic loss of ADAR1p150 in adult mice caused a rapid hematopoietic failure, emphasizing the ongoing requirement for ADAR1p150 in vivo. Overall, these results enhance our understanding of the distinct functions of ADAR1 isoforms and their physiological roles in modulating the innate immune response. Most importantly, the generation of the ADAR1p150-specific knockout mouse model provides a valuable tool for further investigating the functional differences between ADAR1 isoforms and their implications in disease pathogenesis.

**What led you to study RNA or this aspect of RNA science?**

During my master's degree studies, I had the chance to work as a microscopist and gain valuable experience in developing advanced fluorescence imaging techniques. While I thoroughly enjoyed this work, I had a strong desire to explore fundamental biology and address research gaps in the field of diseases and health. When I discovered the PhD project description centered around ADAR1 and its impact on the immune system and normal development, I instantly recognized it as the perfect opportunity I had been seeking. Additionally, the intricacy and adaptability of RNA molecules greatly intrigued me. Upon entering the world of RNA, I realized that contributing to the understanding of RNA's role in diseases and its significance for human health would be intellectually stimulating and personally fulfilling.

**Are there specific individuals or groups who have influenced your philosophy or approach to science?**

My philosophy of science has been influenced by my parents, while my approach to science has been shaped by my lecturers and research supervisors. Despite being business professionals unfamiliar with the scientific world, my parents have instilled in me a strong work ethic and a commitment to work. Their dedication has always served as my inspiration, teaching me the importance of loving what you do and investing significant time in it. Hence, I chose to pursue science, the field I am truly passionate about.

Throughout my studies in biomedical science and research endeavors, my lecturers and supervisors have further guided me in practicing and honing my passion. They have shown me how to embrace science wholeheartedly and have provided valuable insights into conducting research. Their mentorship has been instrumental in shaping my scientific approach.

**What are your subsequent near- or long-term career plans?**

In the near term, I am excited to begin my postdoctoral position focusing on translational or therapeutic research with direct applications for patients. Looking further ahead to the long term, my ultimate aspiration is to work for myself and become an independent researcher. I envision taking my own research ideas and developing them into innovative solutions that can be brought to the market. By bridging the gap between academia and industry, I hope to translate scientific discoveries into practical applications that address unmet needs in healthcare.
